# Global Workspace Dynamics: Cortical “Binding and Propagation” Enables Conscious Contents

**DOI:** 10.3389/fpsyg.2013.00200

**Published:** 2013-05-28

**Authors:** Bernard J. Baars, Stan Franklin, Thomas Zoega Ramsoy

**Affiliations:** ^1^The Neurosciences InstituteLa Jolla, CA, USA; ^2^University of MemphisMemphis, TN, USA; ^3^Copenhagen UniversityCopenhagen, Denmark

**Keywords:** consciousness, awareness, attention, voluntary control, global workspace theory, cortex, theoretical biology, brain dynamics

## Abstract

A global workspace (GW) is a functional hub of binding and propagation in a population of loosely coupled signaling elements. In computational applications, GW architectures recruit many distributed, specialized agents to cooperate in resolving focal ambiguities. In the brain, conscious experiences may reflect a GW function. For animals, the natural world is full of unpredictable dangers and opportunities, suggesting a general adaptive pressure for brains to resolve focal ambiguities quickly and accurately. GW theory aims to understand the differences between conscious and unconscious brain events. In humans and related species the cortico-thalamic (C-T) core is believed to underlie conscious aspects of perception, thinking, learning, feelings of knowing (FOK), felt emotions, visual imagery, working memory, and executive control. Alternative theoretical perspectives are also discussed. The C-T core has many anatomical hubs, but conscious percepts are unitary and internally consistent at any given moment. Over time, conscious contents constitute a very large, open set. This suggests that a brain-based GW capacity cannot be localized in a single anatomical hub. Rather, it should be sought in a *functional hub* – a dynamic capacity for binding and propagation of neural signals over multiple task-related networks, a kind of neuronal cloud computing. In this view, conscious contents can arise in *any* region of the C-T core when multiple input streams settle on a winner-take-all equilibrium. The resulting conscious gestalt may ignite an any-to-many broadcast, lasting ∼100–200 ms, and trigger widespread adaptation in previously established networks. To account for the great range of conscious contents over time, the theory suggests an open repertoire of binding^1^ coalitions that can broadcast via theta/gamma or alpha/gamma phase coupling, like radio channels competing for a narrow frequency band. Conscious moments are thought to hold only 1–4 unrelated items; this small focal capacity may be the biological price to pay for global access. Visuotopic maps in cortex specialize in features like color, retinal size, motion, object identity, and egocentric/allocentric framing, so that a binding coalition for the sight of a rolling billiard ball in nearby space may resonate among activity maps of LGN, V1-V4, MT, IT, as well as the dorsal stream. Spatiotopic activity maps can bind into coherent gestalts using adaptive resonance (reentry). Single neurons can join a dominant coalition by phase tuning to regional oscillations in the 4–12 Hz range. Sensory percepts may bind and broadcast from posterior cortex, while non-sensory FOKs may involve prefrontal and frontotemporal areas. The anatomy and physiology of the hippocampal complex suggest a GW architecture as well. In the intact brain the hippocampal complex may support conscious event organization as well as episodic memory storage.

## Introduction

This paper extends the Global Workspace (GW) theory of conscious experience to brain evidence, particularly the role of the cortex and thalamus (Baars, 1988, 2002; Dehaene and Naccache, 2001; Baars and Franklin, 2003, 2007; Dehaene et al., 2006; Shanahan, 2010, 2012; Dehaene and Changeux, 2011; etc.). While cortex and thalamus look separate to the naked eye, they act as an integrated system (Llinas and Pare, 1991; Edelman and Tononi, 2000; Steriade, 2006; Freeman, 2007). A GW approach helps to organize a large body of evidence and yields distinctive predictions^2^.

High quality empirical evidence has increased rapidly in recent years, both for conscious state studies and for conscious contents. State studies typically compare waking to slow-wave sleep, coma, general anesthesia, and the epilepsies. Studies of conscious contents compare conscious vs. unconscious cognition during the waking state, using binocular rivalry, the attentional blink, backward masking, and attentional manipulations. Both conscious and unconscious stimuli trigger sensory volleys that can be traced well into the cortex (Gaillard et al., 2009; Panagiotaropoulos et al., 2012).

Global Workspace theory has been applied to numerous empirical findings (e.g., Baars, 1988, 2002; Madl et al., 2011, 2012). Brain imaging experiments have supported the best-known GW prediction of “widespread integration and broadcasting” (Dehaene and Naccache, 2001). That is, conscious stimuli typically evoke cortical activity that is more widespread, intense, and correlated than matched unconscious stimuli.

Some of the best evidence to date comes from Panagiotaropoulos et al. (2012) building on almost two decades of findings from intracranial recordings in the macaque. They used an experimental technique called “flash suppression,” involving a long-lasting type of binocular rivalry between the conscious (perceived) but not sensory (unperceived) visual channel. This method allows for “contrastive analysis” of conscious vs. unconscious contents with identical stimulus presentation to the two eyes. Previous work from the Logothetis laboratory has shown conscious visual stimulus representation in temporal regions including IT, MTL, and the temporal association cortex. The most recent report extends this to the lateral prefrontal cortex reporting, “neuronal discharges in the majority of single units and recording sites in the LPFC follow the phenomenal perception of a preferred stimulus. Furthermore, visual awareness is reliably reflected in the power modulation of high-frequency (>50 Hz) local field potentials in sites where spiking activity is found to be perceptually modulated.”

While conscious perception appears to be markedly more distributed and in higher signal fidelity as compared to matched unconscious contents, this does not necessarily apply to other varieties of cognition. Unconscious automatic skills and perceptual inferences, parietal ego- and allocentric maps, sensorimotor control and implicit memory retrieval may show accuracy equal to, or greater than matched conscious events. The brain basis for these differences is not well understood.

This paper develops GW dynamics, suggesting that conscious experiences reflect a flexible “binding and broadcasting” function in the brain, which is able to mobilize a large, distributed collection of specialized cortical networks and processes that are not conscious by themselves. Note that the “broadcast” phase proposed by the theory should evoke widespread adaptation, for the same reason that a fire alarm should evoke widespread responding, because the specific needs for task-relevant responders cannot be completely known ahead of time. General alarms are interpreted according to local conditions.

A brain-based GW interacts with an “audience” of highly distributed, specialized knowledge sources, which interpret the global signal in terms of local knowledge (Baars, 1988). The global signal triggers reentrant signaling from receiving networks, allowing for increasingly focused resonance between source networks and receiving networks.

### Dynamic GW vis-à-vis other theoretical proposals

We can widely divide current theories into philosophical and empirically based ones. Some of the philosophical theories are now generating testable hypotheses.

Empirical theories can be divided into “localist” vs. “local-global” types. There are no exclusively global theories, since no one denies the evidence for local and regional specialization in the brain.

Philosophical theories typically aim to account for subjective experiences or “qualia,” a notoriously difficult question. Recently some philosophical perspectives, like “higher order theory” (HOT) have also generated testable proposals about the involvement of brain regions like the prefrontal cortex. However, brain imaging experiments (e.g., Dehaene and Naccache, 2001) have long implicated the frontoparietal cortex in subjective experience.

It is not clear at this time whether philosophically based theories generate novel, testable predictions. However, efforts are under way to test HOT theories. In general, claims to explain subjective qualia are still debated.

Empirical theories tend to start with reliable phenomena (See Appendix A). For example, the limited capacity of conscious perceptual contents has been discussed since the nineteenth century, and continues to amass a large basis in reliable evidence. One of the driving questions of GW theory is how the limited capacity of momentary conscious contents can be reconciled with the widespread access enabled by conscious contents.

Zeki (2001) makes the localist claim that conscious percepts of red objects involve “micro-conscious” activation of cortical color regions (visual areas V3/V4). However, most empirical theories combine local and global activities, as briefly discussed above. It is still possible that momentary events may be localized for 100 ms or less, and that full conscious contents emerge over some hundreds of milliseconds.

The Dynamic GW theory (dGW) is a specific version of the “dynamic core” hypothesis proposed by Edelman and Tononi (2000), and in somewhat different forms, by Edelman (1989) and others. The cognitive basis of the theory was elaborated by Baars (1988) and in subsequent papers with Franklin and others. Additionally, Dehaene, Changeux, and many coworkers have worked out specific, testable experimental models.

Dynamic Global Workspace theory implies a directional signal flow from binding to receiving coalitions. For each conscious event there is a dominant source and a set of receivers, where the propagated signal is interpreted, used to update local processes, and refreshed via reentrant signaling to the source (Edelman, 1989). Conscious sensations arise in a different center of binding and propagation than “feelings of knowing (FOK),” like the tip-of-the-tongue (TOT) experience, as demonstrated by brain imaging studies (Maril et al., 2001). Directional broadcasting of bound conscious contents is one testable distinction from other proposals (Edelman et al., 2011). Supportive evidence has been reported by Doesburg et al. (2009) and others.

Other theories, like Tononi’s mathematical measure of complexity, phi, seem less directional (Edelman and Tononi, 2000). Llinas and Pare (1991) have emphasized the integration of specific and non-specific thalamocortical signaling, and Freeman et al. (2003) have developed a conception of hemisphere-wide signaling and phase changes.

Nevertheless, current local-global theories are strikingly similar. Whether major differences will emerge over time is unclear.

### Dynamic GW as a local-global theory

In 1988 GW theory suggested that “global broadcasting” might be one property of conscious events. Other proposed properties were:

1. Informativeness, that is, widespread adaptation to the novelty of the reportable signal, leading to general habituation (information reduction) of the news contained in the global broadcast. The evidence now supports widespread neuronal updating to novel input.
2. Internal consistency of conscious contents, because mutually exclusive global broadcasts tend to degrade each other. This is a well-established feature of conscious contents, first observed in the nineteenth century and replicated many thousands of times. Binocular rivalry is one well-known example.
3. Interaction with an implicit self-system. Baars (1988) proposed that the observing self is coextensive with implicit frames that shape the objects of consciousness. The notion of an implicit self now has a great deal of psychological evidence in its favor. Lou et al. (2010) have shown that it may involve the precuneus and orbitofrontal cortex. Others have argued for a midline brain system ranging from the PAG to the orbitofrontal cortex.
4. Limited capacity, and an operating cycle of 100–200 ms. The functional reason for conscious limited capacity has not yet received a satisfactory explanation. We will sketch a possible account below.

After almost a century of scientific neglect, many other aspects of consciousness remain to be studied. In recent times two research programs have pursued GW theory (GWT) in detail. A French group led by Dehaene and Changeux have conducted almost two decades of important research, focusing on formal models of specific experimental paradigms that allow close comparisons between conscious and unconscious conditions. This has been the most systematic and focused modeling and testing program on GWT, called “neuronal GW theory” by the authors. It continues to increase in sophistication and empirical reach over time (e.g., Dehaene and Naccache, 2001).

Franklin and coworkers have pursued GWT in terms of a large-scale cognitive architecture by (Baars and Franklin, 2003, 2007; Snaider et al., 2011; Franklin et al., 2012). A series of published papers by this group suggest a role for conscious events in working memory, selective attention, executive functions, FOK, problem-solving, the memory systems and broader biological functions (see below).

Because almost all neural links in the cortico-thalamic (C-T) system are bidirectional, reentrant signaling from receivers to broadcasting sources may quickly establish task-specific signaling pathways, in the same way that a fire department might locate the source of a community-wide alarm, and, then, communicate in a much more task-specific way. Current evidence suggest brief broadcasts, as suggested by the ∼100 ms conscious integration time of different sensory inputs.

### The reportability of conscious events

Scientific theories often begin with intuitively plausible measures, but in time they must explain those measures. “Accurate report” has long been the most widely used behavioral index of conscious experiences (Baars, 1988). All of sensory psychophysics is based on accurate report, but we have no accepted explanation why conscious cognition should be reportable, while unconscious cognition is not. We suggest an answer to this puzzle below.

### Cortex and thalamus

**Figure 1:**
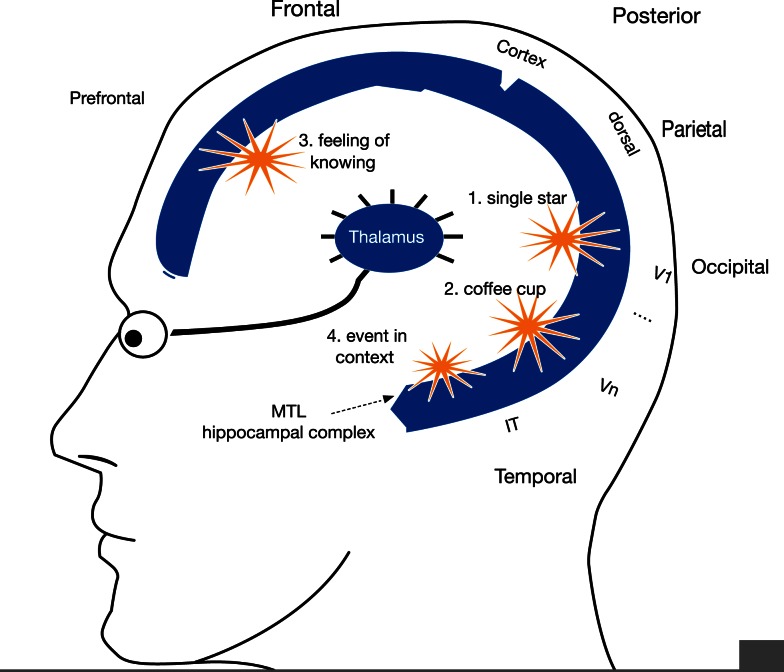
**Binding and broadcasting from many locations**. Four alternative sources of binding and broadcasting in the C-T core. Sites of possible binding and broadcasting are shown as yellow starbursts. Because global broadcasts mutually interfere, only one can occur in any 100–200 ms period. Global interference may explain the limited capacity of momentary conscious contents. Areas V1 and IT: visuotopic maps include area V1, the first cortical map for vision, and area IT, where conscious object representations emerge (Logothetis, 1998). Different coalitions of anatomically identical spatiotopic activity maps may lead to different conscious experiences, like the sight of a single star on a dark night (V1) vs. a coffee cup within arm’s reach (IT). Prefrontal cortex: non-sensory “feelings of knowing” may bind and broadcast from non-sensory cortex. Area MTL: we predict that the intact medial temporal cortex contributes to subjective event organization as well as episodic memory coding (See The Hippocampus and Conscious Contents: a Novel Prediction).

Many brain regions have been proposed to underlie conscious experiences, including the cortex, thalamus, brainstem reticular formation, claustrum, zona incerta, colliculi, prefrontal cortex, visual feature fields, thalamocortical projections, and the like. Recent evidence (e.g., Damasio, 1989; Edelman, 1989; Steriade, 2006; Freeman, 2007; and others) from recordings in the living brain support the “cortex plus thalamus” account for conscious contents in the waking state, at least in humans and other primates (Figure 1). Non-primates may utilize analogous brain structures, like the avian pallium.

This C-T view is based on:

(a) Behavioral and brain imaging studies of cognition during waking compared to sleep, drowsiness, distraction, extended mental effort, sleep deprivation, coma, general anesthesia, and other states.
(b) Direct intracranial stimulation and recording during specific cognitive tasks, like speaking vs. listening to a word.
(c) Empirical dissociations between conscious vs. unconscious brain conditions, like split brain surgery, cortical blindness and parietal neglect. These phenomena can often be replicated in healthy subjects using TMS, hypnosis, drugs, or behavioral manipulations.

The case for the C-T system has recently been strengthened by the use of TMS-EEG, a magnetic pulse aimed at a specific cortical location while recording from a neurally linked one (Massimini et al., 2005). For example, TMS applied to the left somatosensory hand region leads to strong EEG pickup in the contralateral hand region, much like a conductance meter in an electrical circuit. TMS-EEG may become the first practical, minimally invasive measure of causal connectivity in cortex. It has been shown to differentiate between conscious waking vs. sleep and coma, and to distinguish behavioral coma from true coma (Rosanova et al., 2012).

Damage to frontoparietal regions or connections may impair the conscious state. Demertzi et al. (2012) and Blumenfeld (2012) have therefore proposed a consciousness system including large regions of frontal and parietal cortex. While this proposal helps to explain waking state impairments, it does not include the classical sensory cortices. However, sensory events are often thought to be the prototypical contents of consciousness.

We suggest below that in the intact brain, sensory regions can drive frontoparietal areas, so that both regions play essential roles. More generally we propose that conscious events may arise anywhere in the C-T complex during waking, and that they may signal many other loci in the C-T core (see Figure 1). This proposed “any-to-many” recruitment is testable with current methods.

In many cases the original binding source specifies the qualitative conscious contents (the qualia), so that visually attributed experiences are expected to be dominantly sourced in visual cortex, while simple sounds arise in auditory regions. As we will see, non-sensory regions of the cortex can give rise to FOKs, which are richly endowed with reportable contents, even in the absence of sensory cues.

In this view conscious experiences reflect flexible “binding and broadcasting” in the C-T complex, interacting with subcortical, striate and cerebellar regions. GW dynamics (dGW) emphasizes the range and flexibility of binding and broadcasting. It may also help to explain the feats of functional adaptation that can occur after severe cortical damage (see Voluntary Reports of Conscious Events).

Here we aim to preserve the simplicity of the theory while taking into account a wider range of evidence.

Cortex and thalamus consist of multi-layered two-dimensional arrays of neurons and their connective arborizations. Each array projects topographically to others. Under the cortical mantle, white matter pathways run in all canonical directions. These long pathways are targeted in very precise and regular topographies. Local cortical links are also very numerous. Tractographic studies indicate that the connectivity of the C-T system follows small-world network properties (Sporns and Honey, 2006).

The C-T core constitutes by far the biggest parallel-interactive structure in the mammalian brain, showing massive wave-like activities during active states (Steriade, 2006). Most sensory pathways flow into the C-T system, while motor pathways flow outward for subcortical, craniospinal, and autonomic control.

Hundreds of specialized regions of cortex have been identified, and new ones continue to be discovered. Cortical regions are mirrored in major thalamic nuclei, most of which are also layered arrays. Sherman and Guillery (2011) note that “any new information reaching a cortical area… benefits from a thalamic region.” Thalamic nuclei do not communicate with each other directly, but only by way of cortical synapses.

Cortico-thalamic arrays are mutually linked via spatially consistent “labeled lines.” For example, the small foveal center of each retina subtends only 2–4° of visual arc, with about one million densely packed cones and rods. Foveal patches can be roughly considered to be 1000 × 1000 arrays of light receptors that are echoed point-to-point in retinal ganglion cells, whose axons make up the optic nerve. Ganglion cells are mirrored in the visual thalamus (LGN), which transmit signals point-to-point to V1.

Labeled line coding implies that the relative locations of neurons are preserved in higher level retinotopic maps. Beyond V1 visuotopic maps preserve a sparser copy of the visual field, while cellular receptive fields increase in size and decrease in spatial resolution. Spatiotopic mapping is regular and systematic throughout the C-T complex. For instance, there are tonotopic maps in auditory areas, body maps in the somatic regions, and similar such maps in the motor cortex. At higher levels C-T arrays project to self-centered and object-centered spaces, which must be coordinated with each other to support a coherent domain of action and perception. Multiple levels of inhibition serve to regulate and sculpt the excitatory activity of the cortex and its satellites. Cortical arrays therefore resemble the head-up cockpit display of a fighter jet, which may show a geometric frame for the plane itself, another frame for targets, and more for attackers and neutrals. When these floating spatial frames intersect, they may alert executive networks to make decisions.

Starting with LGN, all spatiotopic pathways become bidirectional. Successive arrays pick up visual features like spatial frequency, contrast, edge orientation, gestalt properties, hue, motion, and object identity. Higher level properties like object permanence, size constancy, color constancy, shape from shading, face and object recognition, scene analysis, movements, causality, and event organization, all require complex interactions among 40 or more spatiotopic arrays. The sight of a red traffic light must remain stable in spite of differences in reflectance, observer motion, background clutter, and changes in sunlight.

Figure 1 shows four examples of possible binding and broadcasting in the C-T core (starburst icons). Cortical area V1 and the visual thalamus (LGN) can be conceived as two arrays of high-resolution bright and dark pixels, without color. The sight of a single star on a dark night may therefore rely heavily on V1 and its mirror array of neurons in LGN. V1 and LGN interact constantly, with bidirectional signal traffic during waking.

The conscious sight of a single star at night reveals some surprising features of conscious vision, including spatial context sensitivity, as in the classical autokinetic effect: single points of light in a dark space begin to wander long subjective distances in the absence of spatial framing cues. The autokinetic effect is not an anomaly, but rather a prototype of decontextualized percepts (Baars, 1988). A large literature in perception and language shows scores of similar phenomena, as one can demonstrate by looking at a corner of a rectangular room through a reduction tube that excludes external cues. Any two or three-way corner in a carpentered space is visually reversible, much like the Necker Cube and the Ames trapezoid. Such local ambiguities exist at every level of language comprehension and production (Baars, 1988; Shanahan and Baars, 2005).

The dorsal stream of the visual cortex provides egocentric and allocentric “frames” to interpret visual events in nearby space. These parietal frames are not conscious in themselves, but they are required for visual objects to be experienced at all (Goodale and Milner, 1992). Injury to right parietal cortex may cause the left half of visual space to disappear, while contralesional stimulation, like cold water in the left ear, may cause the lost half of the field to reappear.

Thus even a single dot of light in a dark room reveals the contextual properties of conscious perception. Ambiguity and its resolution is a universal need for sensory systems in the natural world, where ambiguity is commonly exacerbated by camouflage, deceptive signaling, distraction, unpredictable movements, ambushes, sudden dangers and opportunities, darkness, fog, light glare, dense obstacles, and constant utilization of cover by predators and prey (Bizley et al., 2012).

Conscious percepts plausibly involve multiple “overlays,” like map transparencies. The sight of a coffee cup may involve an object overlaid by color, texture, and reflectance, combining information from LGN, V1, V2, V3/V4, and IT (Crick and Koch, 1990). Active cells in those arrays may stream signals across multiple arrays, cooperating, and competing to yield a winner-take-all coalition. Once the winning coalition stabilizes, it may “ignite” a broadcast to other regions.

Conscious vision is strikingly flexible with respect to level of analysis, adapting seamlessly from the sight of a single colored dot to the perception of a dotted (pointillist) painting. An account of conscious vision must therefore explain how a local dot can be perceived in the same visual display as a Georges Seurat painting^3^.

To identify a single star at night, because the highest spatial resolution is attained in the retina, LGN, and V1, the visual cortex must be able to amplify neuronal activity originating in LGN-V1 through attentional modulation. For coffee cups and faces, the relative activity of IT and the fusiform gyrus must be increased. It follows that binding coalitions of visual activity maps can bring out the relative contribution of different feature levels, even for the same physical stimulus (Itti and Koch, 2001).

### Bidirectional pathways and adaptive resonance

Because C-T pathways are bidirectional they can support “reentrant signaling” among topographically regular spatial maps. The word “resonance” is often used to describe C-T signaling (Wang, 2001). It is somewhat more accurate than “oscillation,” which applies to true iterative patterns like sine waves. Edelman and coworkers prefer the term “reentry,” while others, like use “adaptive resonance.” We will use the last term to emphasize its flexible, selective, and adaptive qualities.

**Figure 2:**
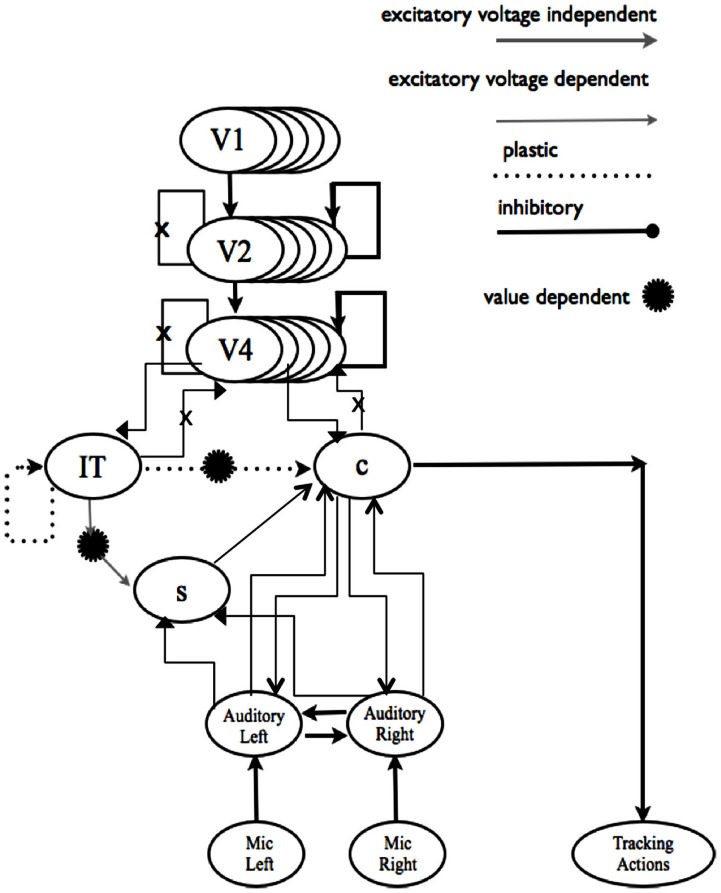
**Feature binding by adaptive resonance**. Darwin VII’s biologically based visuotopic arrays employ adaptive resonance (reentry) to transform optical input into neuron-like activation patterns. Darwin VII was able to bind visual features given the known connectivity of visual cortical areas V1, V2, V4, and IT. Some stimuli have positive or negative “value” (incentives). The robot has goals and movement controls, and perceives obstacles. Thus, the robot’s environment helps to constrain its behavior using goals, rewards, obstacles, and the like.

Adaptive resonance has many useful properties, as shown in modeling studies like the Darwin autonomous robot series, where it can account for binding among visual feature maps, a basic property of visual perception (Figure 2) (Izhikevich and Edelman, 2008). Edelman has emphasized that reentry (adaptive resonance) is not feedback, but rather evolves a selectionist trajectory that can search for solutions to biologically plausible problems. Grossberg and others have developed adaptive resonance models for cortical minicolumns and layers.

A variety of high-level wave-like phenomena emerge in the C-T system, including standing and traveling waves, spiral vortices, centrifugal propagation, phase coupling and decoupling, microstates, cross-frequency coupling, and hemisphere-wide phase transitions at theta-alpha rates (Freeman et al., 2003; Izhikevich and Edelman, 2008). Complex waveforms in the core range from 0.1 to 200 Hz, with momentary spikes up to 600 Hz.

The basic unit of the C-T is therefore not the single neuron, nor the traditional one-directional sensory pathway. It is rather “a unit of adaptive resonance,” which can be thought of as an artificial neural network with at least two layers. Bidirectionality makes the C-T core different from the cerebellum and basal ganglia, which do not support conscious contents directly. Damage to the cerebellum does not directly impair conscious contents, though it can devastate fine motor control. The cerebellum has parallel modules and pathways comparable to a computer server farm, while the C-T complex is parallel-interactive, so that any array of neurons can signal any other. While the cerebellum is more “cognitive” than previously thought, it does not directly enable conscious contents.

Most C-T activity is endogenous: “the cortex talks mostly to itself.” With a few exceptions thalamic nuclei do not communicate with each other directly, but are driven by cortical regions. Steriade (2006) concluded, “*The cerebral cortex and thalamus constitute a unified oscillatory machine displaying different spontaneous rhythms that are dependent on the behavioral state of vigilance*.” (Italics added.)

This is not to minimize the role of neuronal spike timing, which is known to evoke synaptic plasticity. The C-T core may use multiple neuronal codes, including non-classical propagation via glial cells, electromagnetic field induction, electrical synapses, and even membrane ion currents. In conventional terms, brain rhythms interact with single cell activity. Population rhythms require interacting single cells that are both excitatory and inhibitory. Regional rhythms like theta can also recruit single cells that phase-adapt to the peak of the regional wave (Canolty et al., 2010).

Thus neural coding is simultaneously spatial and temporal. The C-T nexus appears to be a vast, parallel-interactive signaling medium, capable of local, regional, and global processing. Tononi has proposed that only the C-T complex combines high integrative capacity within perceived events along with high differentiation between them.

Bidirectional signaling between linked arrays supports many kinds of emergent signaling, just as random Brownian motion allows for many kinds of acoustical wave propagation. Izhikevich and Edelman (2008) have shown that an accurate neuronal simulation of the cortex gives rise to a range of macroscopic waveforms from <0.1 to 200 Hz. Many oscillatory phenomena have been observed in the cortex, including traveling waves, 4–7 Hz microstates, vortices, and centrifugal propagation (Freeman, 2009).

### Feature and frame binding

Visual features are stimulus properties that we can point to and name, like “red,” “bounded,” “coffee cup,” “shiny,” etc. Feature binding is a well-established property of sensory perception.

There is much less discussion about what we will call “frame binding,” which is equally necessary, where “frames” are defined as visual arrays that do not give rise to conscious experiences, but which are needed to specify spatial knowledge within which visual objects and events become conscious. Powerful illusions like the Necker Cube, the Ames trapezoidal room, the railroad (Ponzo) illusion are shaped by unconscious Euclidian assumptions about the layout of rooms, boxes, houses, and roads.

Unconscious assumptions that shape conscious features were previously called “contexts” (Baars, 1988). Because that term is now used with other meanings in the psychological literature, we will adopt to word “framing,” which is in common use in the social sciences. The best-known brain examples are the egocentric and allocentric visuotopic arrays of the parietal cortex. When damaged on the right side, these unconscious visuotopic fields cause the left half of objects and scenes to disappear, a condition called hemi-neglect. Goodale and Milner have shown that even normal visuomotor guidance in near-body space may be unconscious. In vision the dorsal “framing” stream and “feature-based” ventral stream may combine in the medial temporal cortex (MTL) (Shimamura, 2010, see The Hippocampus and Conscious Contents: a Novel Prediction below). Baars (1988) reviewed extensive evidence showing that unconscious framing is needed for normal perception, language comprehension and action planning. In sum, normal conscious experiences need both traditional feature binding and frame binding (Shanahan and Baars, 2005)^4^.

### Consciousness enables many kinds of access (Figure 3)

Animals live in a world of unknowns, surrounded by dangers, and opportunities that may be fleeting, hidden, camouflaged, surprising, deceptive, and ambiguous. Conscious brains may have evolved to cope with such unknowns (Baars, 1988, 2002; Baars, 1988). Newell and colleagues built the first GW architecture to perform acoustical word recognition, at a time when that task was largely underdetermined (Newell, 1990). Their solution was to build a computational architecture, a blackboard model, which would allow many incomplete sources to compete and cooperate to resolve some focal ambiguity. The result was remarkably successful for its time in recognizing nearly 1,000 ordinary words spoken in normal acoustical spaces, complete with hard echoing surfaces, mumbling speakers, and soft, absorbent surfaces, background noises, and the like. Speech recognition is now handled with improved formant tracking, but even today, if semantic unknowns arise in a spoken word stream, a GW architecture may be useful to find the answer. We have no semantic algorithms that interpret word ambiguities across many domains, the way humans routinely do.

**Figure 3:**
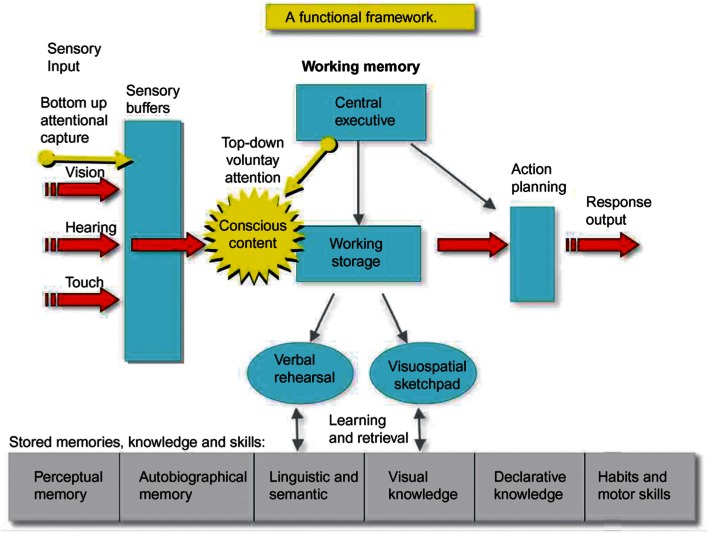
**Conscious contents enable access to cognitive functions, including sense modalities, working memory, long term memories, executive decisions and action control**. Executive regions of the frontoparietal cortex gain control over distributed unconscious functions via conscious feedback. (Cerf et al., 2010; Shanahan, 2010).

Baars and Franklin (2003) used GW theory to propose that consciousness enables access between otherwise separate knowledge sources. One major kind of access that has been discussed since Emmanuel Kant is the access of the “observing self” to the contents of consciousness. Lou et al. (2010) have shown that self-related brain regions like the precuneus and midline structures from the PAG to orbitofrontal cortex may be mobilized by conscious sensory contents. Baars (1988) proposed that self-other access is a specific variety of framing (contextualizing), and that it is a necessary condition for conscious contents.

Global workspace architectures can also “call” fixed automatisms. For example, in speech recognition word ambiguity may be resolved by a known syntactic rule. A global broadcast of the ambiguous word may recruit routines whose relevance cannot be known ahead of time. We have referred to this as contextualization or frame binding (Baars, 1988; Shanahan and Baars, 2005,^5^,^6^,^7^). The “frame problem” is a recognized challenge in artificial intelligence and robotics, but it applies equally to living brains.

Animals and plants co-evolve both accurate and deceptive signals, as in the case of monkeys, bees and flowers. In such an evolutionary context the need to deal with ever-changing unknowns may result in a constant adaptive pressure toward certain kinds of brains. The C-T system occupies 80% of the cranial volume in humans, mostly connective pathways between thin layers of neuronal cell bodies. This costly organ must somehow improve adaptive fitness (Baars, 2002). Specialized algorithms like spinal reflexes are crucial, but in the face of constantly evolving unknowns, a GW architecture may provide a competitive edge. Here, we consider how such a flexible adaptive capacity may be expressed in the brain.

### Spatiotopic activity maps, streams, and coalitions

Spatiotopic organization is common in the C-T core, including the tonotopic maps of the auditory system, multimodal maps of parietal cortex, and visual maps of the prefrontal cortex. Both sensory input and neuromuscular output use spatial arrays. The senses also converge on abstract spatial representations.

Thus the concept of a spatiotopic activity map (SA map) is useful, defined as a pattern of neuronal activity over a spatiotopic array of cells. We start with “activity maps” rather than structural maps, because only a subset of the cells in any spatiotopic array is active at any given moment. Therefore cortical and thalamic layers can be treated as four dimensional matrices with indices (*i*, *j*, *k*, *t*), with positive or negative scalars for excitatory, or inhibitory cells at time *t*. Active neurons can then be labeled CELL#<*i*, *j*, *k*, *t*> and take on numerical values above or below a threshold during some brief time period, perhaps 0.1 s, since neurons in cortex may fire at a baseline 10 Hz rate. While this is highly simplified, it leads to a sensible conception of a brain-based GW.

Figure 2 can be viewed as SA maps that combine with others into SA streams; SA streams may compete or cooperate until some winner-take-all coalition results in a stable outcome, perhaps as briefly as the peak of the alpha or theta wave (Freeman, 2007). Together, the many active layers of the visual cortex can act as a resonant dynamical system that can yield an open set of visual experiences over time.

### Any-to-any signaling

**Figure 4:**
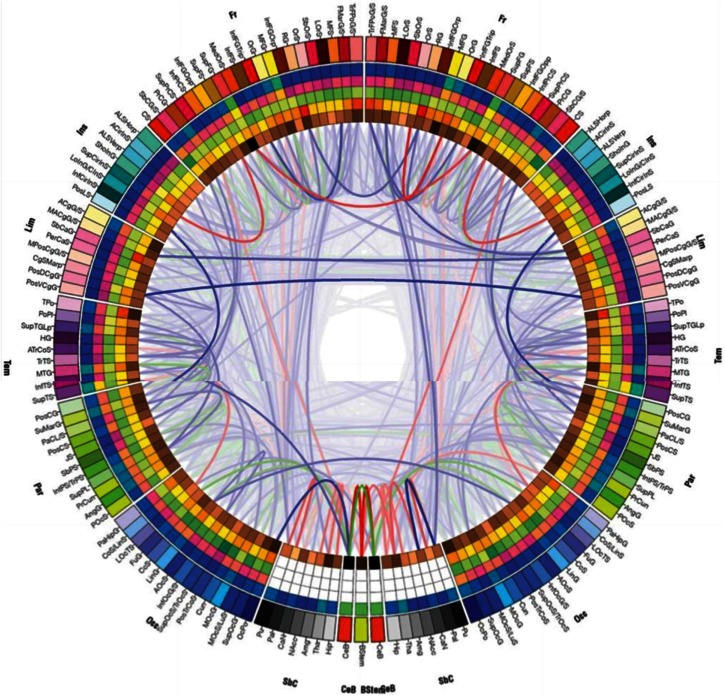
**The C-T complex supports any-to-any signaling**. The cortico-thalamic system allows any spatiotopic activity (SA) array to signal any other. Combined with adaptive resonance, this allows an open set of cortical and thalamic coalitions to bind and broadcast information from any region to any other. The left half represents the left hemisphere of the brain, whereas the right half represents the right hemisphere. The brain stem is shown at the bottom. Circular color bars at the bottom describe the scale of the corresponding anatomical ring.

Figure 4 shows the major long-distance connections in the C-T system of the macaque, including the basal ganglia (Modha and Singh, 2010). It is consistent with the notion of “any-to-any” signaling among SA arrays in cortex, mirrored in thalamic nuclei. Any cortical array can signal any other, both directly and via thalamic relays.

Modha and Singh also emphasize an “inner core” of high-density connections, consistent with a Neural Darwinist perspective of synaptic growth and myelination by use. In effect, the white matter pathways of the C-T system should reflect their traffic density, much as highway engineers would broaden highways to reflect their maximum usage, while allowing low-density access to residential streets. High-resolution tractography of the white matter pathways shows small-world network properties, consistent with efficient allocation of neuronal traffic flow (Achard et al., 2006).

**Figure 5:**
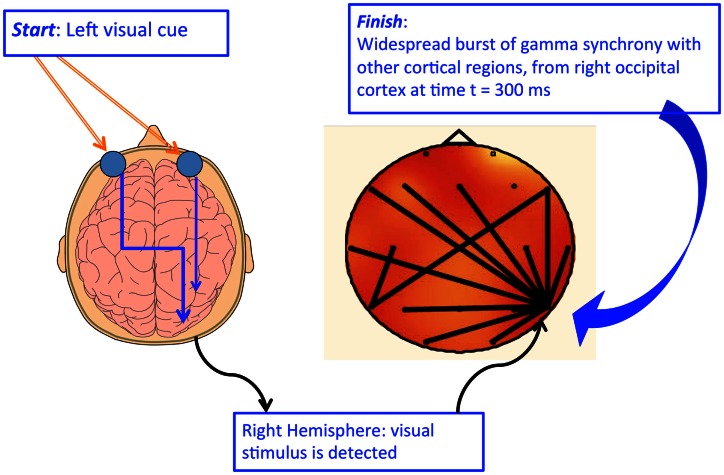
**Any-to-many signaling in conscious vision**. While the structure of the cortico-thalamic system suggests any-to-any signaling, effective (causal) connectivity requires measures of signal traffic flow rather than structural connectivity. Doesburg et al. (2008) were able to show “any-to-many” signaling using simple LED stimuli in the lateral periphery of each hemifield. The figure shows a left-lateralized light stimulus triggering a broadcast from early right visual cortex.

**Figure 6:**
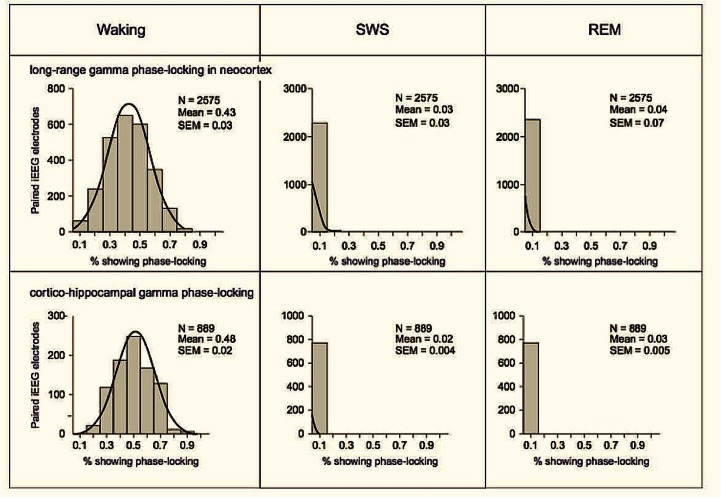
**Long-distance phase-locking in the waking state**. The waking state shows hidden regularities. During waking, intracranial EEG shows phase-locking between different parts of cortex, and between the hippocampus and neocortex. While the overall sound of the waking stadium may seem random, local conversations can show precise phase coupling. Notice that deep sleep and dreaming show almost no phase coupling between distant cortical regions, or between cortex and hippocampus.

In the visual cortex, dynamic SA coalitions may represent a single star on a dark night, a coffee cup on a nearby table, a simple event such as a cup falling off a table, and the textured surfaces of visual objects. Using the rich set of connectivities shown in Figure 4, such a stable visual coalition can signal to other C-T regions. The C-T system enables phase coupling and decoupling among active spatiotopic arrays (see also Figures 5 and 6). Steriade (2006) suggests a hierarchy of cortical oscillations ranging from <1 to 200 Hz, able to partly depolarize large numbers of neurons.

The functional anatomy of Figure 4 suggests a capacity for “any-to-any” signaling among SA maps, streams, and coalitions. Other authors report that C-T connectivities can be described by small-world mathematics (Sporns and Honey, 2006), and Tononi’s formal measure phi provides a global index of connectivity in the C-T core. Such arrays of activity maps can also represent shifting attentional focus in the visual cortex (Itti and Koch, 2001).

SA coalitions follow trajectories that evolve by adaptive resonance. Edelman (1989) has made the case for genetic, ontogenetic, and finally moment-to-moment selectionist processes in the brain. Each selectionist stage shows replication, variation, and selection, including the moment-to-moment dynamics of the C-T core. Dynamic activity travels along pathways and synapses built by use, which are constantly being pruned, beginning in the first month *in utero*.

Entire visuotopic arrays can play inhibitory or excitatory roles, so that SA maps can take on all positive or all negative values. This is useful when the visual system needs to focus on a single level of analysis, like a single colored dot in a Seurat painting, rather than a gestalt of many colored dots.

### Broadcasting: any-to-many signaling

A few ants can secrete alarm pheromones to alert a whole colony to danger, an example of any-to-many broadcasting among insects. In humans the best-known example is hippocampal-neocortical memory storage of memory traces in the neocortex by way of the hippocampal complex (Nadel et al., 2000; Ryan et al., 2001). Memories of conscious episodes are stored in millions of synaptic alterations in the neocortex (Moscovitch et al., 2005). We will return to this point in section “Voluntary Reports of Conscious Events.”

Computer users are familiar with global memory searches, which are used when specific searches fail. The C-T system may enable brain-based global memory searches. “Any-to-many” coding and retrieval can be used to store and access existing information (Nadel et al., 2000; Ryan et al., 2010). It is also useful for mobilizing existing automatisms to deal with novel problems.

Figure 4 shows how conscious visual stimuli may trigger a burst of any-to-many signaling from the right visual cortex to both hemispheres. By instructing subjects to attend to a lateralized right or left stimulus while keeping the eyes physically fixated on a target, either the right or left LED can be made conscious.

Notice that “any-to-many” signaling does not apply to the cerebellum, which lacks parallel-interactive connectivity, or to the basal ganglia, spinal cord, or peripheral ganglia. Crick and Koch have suggested that the claustrum may function as a GW underlying consciousness. However, the claustrum, amydgala, and other highly connected anatomical hubs seem to lack the high spatiotopic bandwidth of the major sensory and motor interfaces, as shown by the very high-resolution of minimal conscious stimuli in the major modalities. On the motor side there is extensive evidence for trainable voluntary control over single motor units and more recently, for voluntary control of single cortical neurons (Cerf et al., 2010). The massive anatomy and physiology of cortex can presumably support this kind of parallel-interactive bandwidth. Whether structures like the claustrum have that kind of bandwidth is doubtful. The sheer size and massive connectivity of the C-T system suggests the necessary signaling bandwidth for a human being to see a single near-threshold star on a dark night.

We do not know the full set of signaling mechanisms in the brain, and any current model must be considered provisional. Neural computations can be remarkably flexible, and is, to some degree, independent of specific cells and populations. John et al. (2001) has argued that active neuronal populations must have dynamic turnover to perform any single brain function, like active muscle cells. Edelman and Tononi (2000) and others have made the same point with the concept of a dynamic core. GW capacity as defined here is not dependent upon the mere existence of anatomical hubs, which are extremely common. Rather, it depends upon a dynamical capacity, which operates flexibly over the C-T anatomy, a “functional hub,” so that activated arrays make up coherent “coalitions.”

The global neuronal workspace has been used to model a number of experimental phenomena. In a recent model, sensory stimuli mobilize excitatory neurons with long-range cortico-cortical axons, leading to the genesis of a global activity pattern among workspace neurons. This class of models is empirically linked to phenomena like visual backward masking and in attentional blindness (Dehaene and Changeux, 2005).

An advantage of the GW approach is that it is clearly functional; that is, it describes a known architecture for integrating and distributing focal information in a large-scale, massively parallel, brain-like organ. This functional architecture has been used for practical applications since Newell and colleagues developed it in the 1970s. However, it is a major challenge to scale neuronal models to the level of real brains. Current neuronal GWT models make use of simplified network architecture.

Franklin et al. (2012) have combined several types of computational methods using a quasi-neuronal activation-passing design. High-level conceptual models such as LIDA can provide insights into the processes implemented by the neural mechanisms underlying consciousness, without necessarily specifying the mechanisms themselves. Although it is difficult to derive experimentally testable predictions from large-scale architectures, this hybrid architecture approach is broadly consistent with the major empirical features discussed in this article. It predicts, for example, that consciousness may play a central role in the classic notion of cognitive working memory, selective attention, learning, and retrieval.

## States and Contents

### Waking and deep sleep

In mammals all goal-directed behavior occurs in the waking state, which enables fast, flexible, and widespread processing. State differences can be seen with the naked eye in the scalp EEG, which measures the post-synaptic activity of large numbers of cortical pyramidal neurons whose axons run orthogonal to the scalp. However, scalp recordings reflect only 0.1% of the cortical voltage, so that intracranial recordings give a much better picture.

Waking EEG looks irregular, complex, and low in amplitude, while deep sleep is marked by regular, simpler, and high-amplitude waves, reflecting coordinated “buzz-pause” firing among billions of neurons near 1 Hz. REM dreaming looks similar to waking, and dreamers often report rich conscious experiences.

Spontaneous conscious mentation occurs throughout the waking state, reflecting repetitive themes described as “current concerns”. Conscious mentation is also reported when subjects are awoken from REM dreams and even from slow-wave sleep. The last may reflect waking-like moments during the peaks of the delta wave (Valderrama et al., 2012).

Thus the waking state appears to enable brain-wide, adaptive resonance in the cortex and thalamus. By comparison, slow-wave sleep – the least conscious state of the daily cycle – may interrupt processing due to massive firing pauses in the trough of the delta wave (Edelman and Tononi, 2000).

Both sleep and waking are under precise homeostatic state control (John et al., 2001), controlled by thalamocortical circuits timed by basal brain nuclei. More than 200 types of epigenetic expression accompany sleep and waking in rats, suggesting many basic biological functions. Sleep appears to be a survival necessity, since rats die from complete deprivation after only 3 weeks. The exact reason is still unknown.

### Global chatting, chanting, and cheering

Global brain states can be compared to a football crowd with three states, “chatting,” “chanting,” and “cheering.”

Chatting describes the C-T activity of waking and REM dreams. It involves point-to-point conversations among spatial arrays in the C-T system, which can have very high S/N ratios, though they appear to be random when many of them take place at the same time. Like a football stadium with thousands of coordinated local conversations that are not coordinated globally, the average global activity is a low-level crowd roar, seemingly random, which appears to be fast and low in amplitude. Nevertheless, as we will see, direct cortical recordings show phase-coupled chatting in the C-T core appears to underlie specific cognitive tasks. Thus chatting activity gives the misleading appearance of randomness en masse, but it is in fact highly organized in a task-driven fashion. Because sports arenas show the same properties, the arena metaphor provides us with a useful reminder.

Chanting shows coordinated start-stop crowd activity, about once a second over a prolonged period of time, like the “buzz-pause” rhythm of billions of neurons in the C-T core, which results in global delta waves. Chanting sounds like chatting at the peak of the delta wave, followed by simultaneous pausing, which interrupts all conversations at the same time (Massimini et al., 2005). Breakdown of cortical effective connectivity during sleep.

Deep sleep (also called delta sleep or slow-wave sleep) is the least conscious state of the daily cycle, perhaps because 1–4 Hz pauses during the DOWN hemicycle of delta disrupt any cognitive tasks. However, there is some reported mentation when people are awoken from delta sleep, suggesting that the peak activities may be conscious or preconscious, so that during the process of awakening the chanting state is interpreted by the cortex in coherent ways. Because we cannot get instant reports from sleep, it is not obvious whether the reports are shaped by the seconds it takes to wake up.

Finally, a stadium crowd may cheer when a team scores a goal or makes an error. This corresponds to an “event-related” peak of activity. In the brain, the event-related potential (ERP) occurs when a significant or intense stimulus is processed, causing a stereotypical wave pattern to sweep through the brain. The event ERP shows a series of negative and positive deviations over a period of ∼600 ms, corresponding to early sensory processing (N100), stimulus recognition, and attentional modulation (P200), decision making, and mismatch detection (P300 a and b), and stimulus meaning (N400 and longer). Revonsuo and colleagues have found that a conscious visual stimulus contributes a small negativity near the P3b wave (see Figure 8). ERP peaks and valleys are sensitive to numerous cognitive variables, but their overall shape tends to remain stable. ERP waves show both stimulus-triggered resetting and ongoing background activity.

In section “States and Contents” we suggested that the ERP reflects a repeating perception-action cycle that is mostly unconscious, but which embeds conscious moments lasting around 100 ms. The extended LIDA model interprets both conscious and non-conscious components of such “cognitive cycles” (Franklin et al., 2012).

Using direct brain recording in humans, Figure 6 shows that the waking state enables fast, long-distance phase-locking in the C-T core. Phase-locking is measured by lagged correlated activity among widely dispersed electrodes, and is believed to represent signaling among the cortical arrays being recorded. In this case the signal is measured in the low gamma band, 35–58 Hz, but strikingly similar findings are common from <1 to 200 Hz. Delta, theta, alpha, beta, and gamma phase coupling are commonly reported for a variety of tasks. This very wide band of apparently functional signaling is being studied intensively.

Phase coupling in the gamma range is tightly linked with the waking state. Slow-wave sleep and REM dreaming show a drastic drop in array-to-array gamma coherence. Observed gamma synchrony may reflect underlying theta-gamma cross-frequency coupling, which may in turn be carried by Slow Oscillation-theta-alpha coupling, as suggested by Steriade (2006). Slow Oscillations (SOs) continue throughout the daily cycle at <1 Hz. Widespread SO’s may change the firing threshold among billions of neurons, just as regional theta activity might do at a faster rate. A slow-to-fast wave hierarchy is one way to coordinate large populations of neuronal oscillators in the brain.

### Task-related frequency coupling

A conscious task tends to recruits many cortical and subcortical populations. A range of evidence suggests that phase-coupled firing of large numbers of neurons is involved. Gamma and alpha-range synchrony are implicated in the conscious interpretation of ambiguous stimuli. However, alpha and theta activity in large populations of cells has been observed in other tasks as well. These ∼10 Hz oscillations may serve to group faster rhythms such as beta and gamma. Both alpha-gamma and theta-gamma phase coupling have been observed.

Direct brain recording suggests that task-specific activity involves cross-frequency coupling at multiple spatial scales, linking, and unlinking multiple sites in the C-T core and it satellites. Single neurons can also self-adapt to regional theta oscillations (Canolty et al., 2010; Canolty and Knight, 2012). Population oscillations and single neuronal spike timing may therefore mutually entrain.

**Figure 7:**
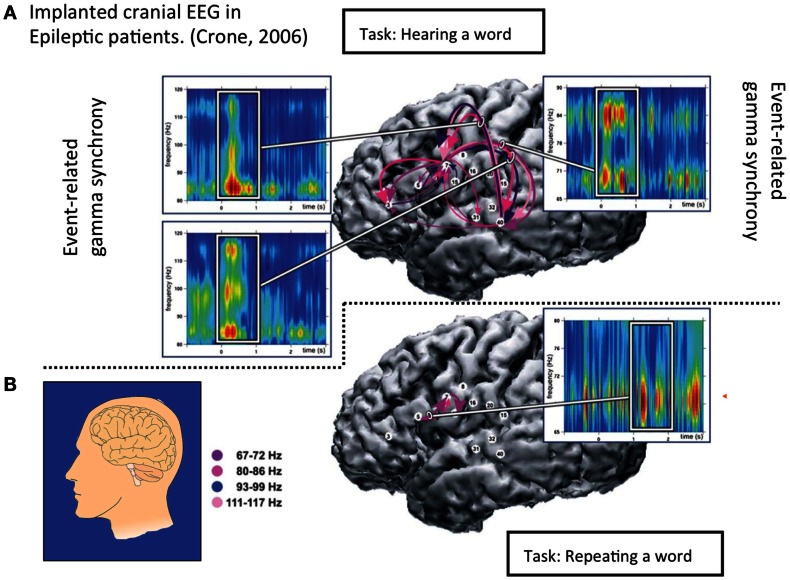
**Intracranial recordings in epileptic patients have 1,000 times the signal-to-noise ration of scalp recordings, and therefore reveal much more detail**. Crone et al. (2006) published these images of the left lateral hemisphere in a conscious epileptic patient before surgery, listening to a spoken word **(A)** and speaking it **(B)**. Conscious patients in this procedure experience little pain under local anesthetic. White numbered disks are electrodes, and purple arrows between them indicate event-related synchrony (ERS) as shown in the graphs. In Task **(A)** “Hearing a word,” ERS bursts in four gamma bands occur 100–600 ms post stimulus. In Task **(B)** “Speaking a word” ERS gamma starts before the response, and continue during the next two seconds in 100–200 ms bursts. Task **(A)** shows widespread left hemisphere ERS, while in Task **(B)** is ERS is localized near Broca’s area for speech production. Other studies show precise ERS bursts in cortex for sensory processing, response organization and memory coding. Cortical synchrony may be a task-specific signaling code.

Figure 7 shows direct cranial recordings from the left hemisphere in an epileptic patient, showing precise place-to-place signaling underlying the gross “random-looking” activity of the waking brain. Alpha-gamma and theta-gamma phase coupling serves to coordinate neurons that may be widely dispersed, but which support similar tasks.

In deep sleep, rapid interactive linking between regions may continue during the “up” sweep of the delta/Slow Oscillation, but it is inhibited during the “down” sweep (Edelman and Tononi, 2000).

### Conscious contents

Traditional psychophysics compares conscious stimuli to each other, a method that dates back to Newton’s prism experiments. However, in a recent wave of research, conscious events are compared to unconscious ones that are physically similar, though they cannot be reported as conscious even under optimal conditions. Many experimental paradigms allow for such comparisons. We can therefore perform two kinds of comparisons for any conscious stimulus: psychophysical differentiation and iso-stimulus contours to define the feature boundaries of the percept and parametric variation to specify the boundaries between conscious and unconscious versions of the same event. When content parameters can be precisely controlled we can therefore make claims about the contents of consciousness during waking, dreaming, twilight states, and even slow-wave sleep.

### A changing stream of bound moments

The flow of conscious moments is often described as a stream (James, 1890). Under careful experimental conditions perceptual events appear stable and internally consistent. The Necker cube is a useful example of a bistable stimulus that is perceived as internally consistent at any given moment. Perhaps all the words of natural language are ambiguous in that they change meaning in different contexts (Baars, 1988). Under normal conditions only one meaning becomes conscious; the process of disambiguation is usually unconscious. The stream of consciousness is therefore constantly changing, but momentary events are perceived as stable and consistent. It is plausible that the stream’s contents emerge from ongoing winner-take-all competitions between activity streams (see Consciousness Enables Many Kinds of Access).

Here we focus on spatiotemporal coding as described above. However, this is a fast-moving frontier. While labeled line topographical resonance is a plausible contributor to the stream of specific conscious experiences, the same mechanism in the dorsal stream does not seem support conscious experiences directly (e.g., Milner and Goodale, 2008). This suggests that we may know some necessary but not sufficient conditions for conscious contents (viz., Baars, 1988). There are several other proposals for the ways in which conscious events may be encoded in cortical activity (Freeman, 2007). The full set of conditions is not yet known.

### Conscious and unconscious task elements

**Figure 8:**
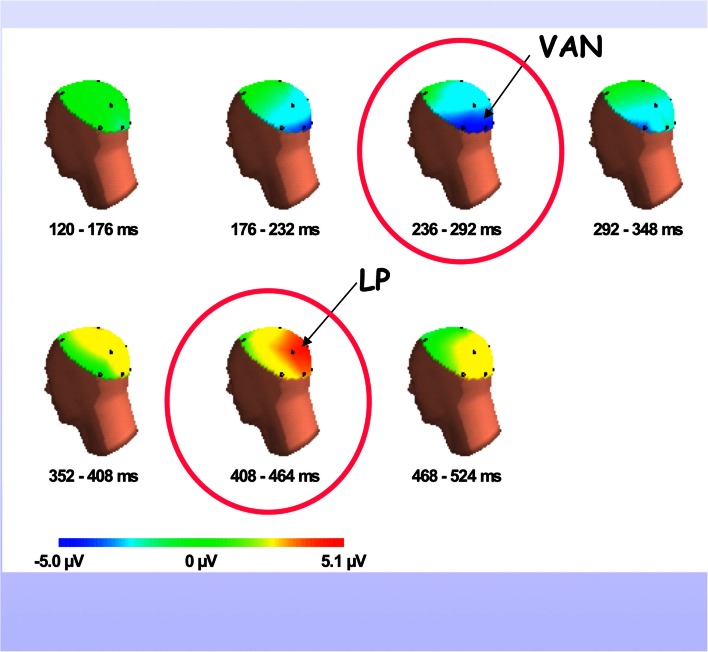
**VAN: the visual awareness negativity wave**. The small conscious content-linked component of the visual awareness ERP (VAN) appears about 300 ms after a visual stimulus and lasts for 1–200 ms. It appears to sweep forward, and may be followed by a late positivity (LP). The VAN has been observed in more than a dozen studies comparing conscious to unconscious visual stimuli that are identical at the retina.

Efficient cognitive tasks optimize tradeoffs between conscious and unconscious, and consciously mediated information processing. As described above, the ERP is a stereotypical waveform lasting about 600 ms, triggered by an intense or significant stimulus, and usually averaged over trials to allow common population responses to emerge while unrelated activity averages to zero. However, it has been difficult to separate the conscious and unconscious aspects of the ERP. New evidence shows that conscious (compared to unconscious) visual stimuli trigger a small difference in the visual ERP, emerging as a Visual Awareness Negativity (VAN) near the conventional P3b waveform, ∼300 ms after stimulus onset and lasting for 100–200 ms (Gaillard et al., 2009). The VAN is believed to sweep forward from occipital-temporal regions (see Figure 8). These results have been replicated more than a dozen times, with different techniques. They suggest that the distinctively conscious signal adds only a small energy component to unconscious background processes, even though they may be triggered by the identical sensory event.

While this may seem surprising given the subjective richness of conscious events, it is not necessarily different from other kinds of signal processing. The most obvious example is radio waves, which use far more energy to propagate carrying waves than they do for the contents of interest, which only modulate the carrier wave. While analogies must be treated with care, there is evidence that the intrinsic activity of the C-T core outweighs external input.

Thus cortical activity as shown in the visual ERP does not determine conscious contents directly. For example, the “novelty P3b” wave reflects population responses to unexpected events, which are processed unconsciously, but which may lead to conscious percepts. The P3 a and b components may therefore reflect preconscious processes. As suggested, the stereotypical ERP may represent a perception-cognition-action cycle in which a global broadcast “ignition” is only one brief component (see Sensory percepts vs. feelings of knowing below). Figure 8 shows the consciousness-linked ERP component emerging in occipital cortex between 200 and 300 ms post stimulus, and followed by a late positivity (LP).

As pointed out, the C-T complex also engages subcortical satellites like the cerebellum and basal ganglia, which do not determine conscious contents directly.

### Winner-take-all coalitions: Microstates

**Figure 9:**
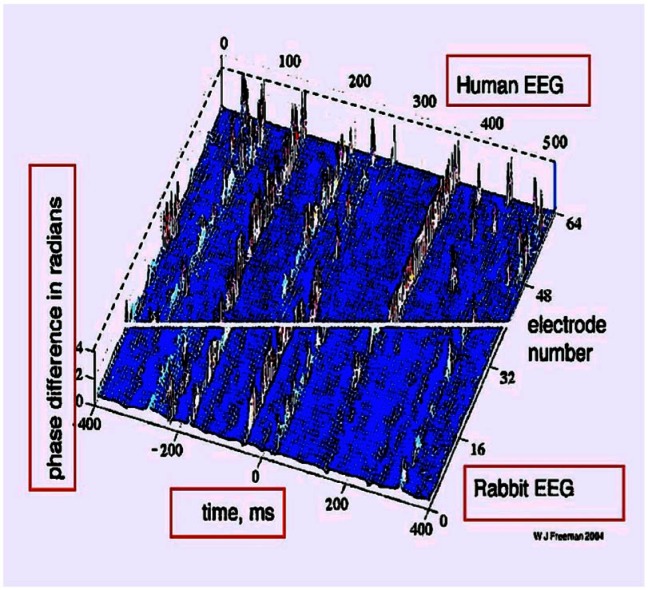
**EEG microstates at theta rates in humans and rabbits**. Global microstates have been found using several methods. Freeman et al. (2003) used Hilbert analysis to observe fast phase changes at theta rates in entire cortical hemispheres in humans and rabbits. The vertical axis shows phase differences in radians, revealing stable states for 100–200 ms, interrupted by a hemisphere-wide near–instantaneous collapse for 5–10 ms before a new equilibrium is achieved. The horizontal axes show time (in ms) and electrodes.

Figure 9 shows evidence for stable microstates in the EEG for both the rabbit and humans, exhibiting rapidly changing phase after ∼100–200 ms, the rate of theta oscillations (Freeman, 2007). Other laboratories, using quite different methods, have also reported momentarily stable, content-sensitive microstates. The current theory suggests that microstates represent binding and broadcasting equilibria involving dynamic coalitions of adaptively resonant populations of neurons. This view seems quite compatible with sophisticated theoretical work by Freeman and Kozma. Crick and Koch (2005) suggested the term “coalitions” for this general concept.

Coalitions can be linked to conscious (rather than unconscious) stimuli even at the level of single cell activity. In an important experimental series, Logothetis and colleagues recorded directly from visual cortical neurons in the macaque, while presenting rivaling stimuli known to evoke activity from edge detection to visual object identification. Neuronal competition and cooperation appeared in established cortical feature maps, with a decisive majority of neurons firing to the conscious percept only in the visual object area, corresponding to inferior temporal cortex (area IT) in humans. Thus, conscious, but not unconscious, visual stimuli evoked a decisive “vote” of neurons in IT.

Logothetis et al.’s research program continues to yield spectacular results including the strongest direct evidence for global distribution of conscious visual stimulation to date (Panagiotaropoulos et al., 2012). More recent studies indicate involvement of the dorsolateral prefrontal cortex, which also has visuotopic maps. However, the bilateral removal of the prefrontal cortex does not abolish binocular rivalry, suggesting, again, that binding of visual input is not localized in a single region, but varies dynamically depending on stimulus, task, anticipatory set, emotional valence, memory load, distractors, and perhaps cortical resource allocation.

Several laboratories have now reported EEG microstates lasting 100 ms or longer, and correlated with conscious contents. In our terms, microstates show momentary equilibria in the C-T core. Freeman and colleagues provide a sophisticated non-linear dynamical interpretation of microstates. Adaptive resonance among topographically organized arrays leads to a dynamic system trajectory that may (1) land in a local minimum, (2) diverge and dissipate, or (3) even lead to a destructive positive feedback loop. Positive feedback among excitatory neurons resembles an epileptic seizure, and large-scale simulations of the cortex do show such activity. However, excitatory activity is constantly regulated by local, regional, and global inhibition, keeping the resonant trajectory between dysfunctional boundary states. Over time, adaptive resonance therefore either disappears, or it may evolve a temporary but stable equilibrium.

Since “binding” and “broadcasting” involve adaptive resonance, the distinctive type of signaling in the C-T core, dGW suggests “binding resonance” to define the winner-take-all gestalt that becomes conscious and “broadcasting resonance” to propagate the winning gestalt to receiving networks.

A human analogy for broadcasting resonance would be a college class equipped with feedback clickers to respond to points made by a lecturer. Signaling goes in both directions, and clickers may be used to ask the lecturer to explain some point in greater detail, so that signal receivers can influence the duration of the broadcast. Broadcasting leads to widespread adaptation among receiving processors (Baars, 1988, see Sensory Percepts vs. Feelings of Knowing); by analogy, a lecture leads to widespread learning. If students need more time to understand a broadcast, they can use their clickers to keep the broadcast signal flowing until they can adapt to it.

The most interesting possibility is that reported microstates reflect both binding and broadcasting in the brain, much like double-wing Lorenz attractors. Using a Hilbert analysis of the cortical EEG, Freeman et al. (2003) report that electrophysiological equilibria reflect a hemisphere-wide spatial phase change, occurring at theta rates. Future work will explore this link.

Recent evidence shows that single neurons can phase-tune their firing to regional theta oscillations, allowing single cells to coordinate with population activity (Canolty et al., 2010). Single cortical neurons can also come under voluntary control (Cerf et al., 2010), suggesting that even single units can serve executive goals.

### Conscious moments are embedded in cycles

The stereotypical ERP spins out over some 600 ms, reflecting basic population activities in processing incoming signals. Conscious moments are believed to last only 100–200 ms, and their distinctive signal appears to be small (Koivisto and Revonsuo, 2007). Franklin et al. (2012) have suggested that binding and global broadcasting is only one part of the recurrent action-perception cycle (Fuster, 2004). While conscious moments lasting ∼100 ms may interfere with each other, the unconscious or automatic half-second of the cognitive cycle may overlap. Automatic skill components interfere less with each other than conscious contents.

Conscious binding and broadcasting seems to be largely a cortical-thalamic event that may trigger output to the striatum of the basal ganglia, a major output hub, which loops back to thalamus and cortex, providing a pathway for a recurrent unconscious cycle (Cole and Schneider, 2007).

## Sensory Percepts vs. Feelings of Knowing

James (1890) emphasized that conscious contents are not just sensory; they include what he called “fringe” experiences or FOK, such as the TOT experience, accurate judgments, concepts (as opposed to percepts), relational terms like “and,” “or,” “but,” vivid expectations, beliefs, and other non-sensory but reportable events (Table 1). FOK show distinct BOLD activity in the frontal cortex, with fewer sensory maps than posterior cortex (Maril et al., 2001). Frontal cortex impairments also increase vulnerability to word retrieval difficulties (Pannu and Kaszniak, 2005).

**Table 1:** **Subjectively vague feelings of knowing**.

The tip of the tongue	TOT state shows semantic knowledge while searching for a missing word. TOT’s activate prefrontal regions fMRI (Maril et al., 2001*).

Judgments	Judgments of beauty, goodness, moral value, confidence, familiarity, rightness, etc., lack perceptual features and shapes, figure-ground contrast, specificity in space, time and content, etc. In contrast, conscious percepts are always particulars.
Relational terms	Prepositions like “to,” “from,” “by,” and relational terms like “above,” “below,” “before,” and “after” are not perceptual, though they may evoke feelings of knowing.

Dynamic Global Workspace theory suggests that FOKs are bound and propagated from non-sensory regions of cortex, such as the classical association areas, frontoparietal regions, and the anterior temporal cortex. Brain imaging suggests that semantic knowledge is distributed in temporal, frontal, and parietal cortex, while sensory regions are recruited for imagery, inner speech, and motor activities that are associated with abstract concepts. However, effortful FOKs appear to be relatively localized in DL-PfC and ACC regions. Effort-related BOLD activity spreads outward as tasks grow more difficult (Tables 2 and 3).

**Table 2:** **A summary of hypotheses**.

Any-to-many binding and broadcasting in the C-T system
Perceptual consciousness vs. feelings of knowing
Dominant coalitions
Task-related chatting via phase coupling and decoupling
Feelings of effort interpreted
Voluntary control involves binding and broadcasting in the prefrontal lobe
Voluntary attention
The relationships to states of consciousness
The generality of adaptive resonance in C-T biocomputation

**Table 3:** **Some testable predictions**.

1. The cortico-thalamic system supports any-to-many binding and broadcasting of conscious contents. A bound conscious gestalt may emerge from *anywhere* in the cerebrum, and spread globally to all other regions for ∼100 ms.
2. Receiving networks adapt to novel information from broadcast sources. After widespread receiver adaptation (updating), broadcasts are driven out of consciousness by competing inputs.
3. Posterior cortex generates perceptual conscious contents, while explicit feelings of knowing are generated from non-sensory cortex. (Frontal, anterior temporal, parietal). Many cognitive tasks involve both a conscious perceptual and a reportable semantic broadcast, as shown in the extended ERP.
4. Since nearly all cortico-thalamic links are bidirectional, the cerebrum supports very widespread adaptive resonance (reentrant signaling). Signaling of conscious contents is superimposed on baseline resonant activity in the C-T core. Because of spatiotopic array organization in the cortex and thalamic nuclei, content signaling in the cerebrum is simultaneously spatial and temporal.
5. Goal-directed signaling in the C-T core is waking state dependent. Waking, dreaming and slow-wave sleep reflect distinct global modes. However, even slow-wave sleep may support waking-like activity during the UP phase of the slow oscillation.
6. While many spatiotemporal codes may exist, cross-frequency phase coupling is thought to integrate the full range of C-T rhythms. Because conscious sensory events are integrated within 100 ms periods, 4–12 Hz rhythms may underlie conscious moments.
7. Effortful voluntary control involves binding and broadcasting from frontoparietal regions. Mental effort is an FOK that is associated with major cognitive styles like persistence and general intelligence.
8. The hippocampal complex supports conscious event perception, as well as serving to encode episodic memory traces in multiple brain regions. Hippocampal lesions often lead to cortical reorganization of conscious sensory functions.

Feeling of knowing are not imprecise in their underlying contents. They are simply subjectively vaguer than the sight of a coffee cup (Baars, 1988), lacking clear figure-ground contrast, differentiated details and sharp temporal boundaries. However, concepts, judgments, and semantic knowledge can be complex, precise, and accurate. The history of science and mathematics is filled with examples of accurate, but subjectively vague, insights that could not be articulated and tested until much later.

Thus, Fermat’s Last Theorem occupied mathematicians for centuries, as a compelling but unproven FOK. Inexplicit but subjectively persuasive FOKs are a recognized route to mathematical discovery. In the brain, semantic knowledge is widely distributed and seems to lack figure-ground contrast.

Tip-of-the-tongue experiences can be induced by asking for the technical names of familiar facts. The question “What are two names for flying dinosaurs?” may elicit strong FOK. Subjects who cannot recall those names still choose accurately and quickly between “pterodactyl” and “brontosaurus.” Semantic knowledge may be fully primed in TOT states, before the lexical form of the missing words can be recalled. Such FOK commonly occur when we have compelling and accurate expectations and intentions. They are not limited to language.

Psycholinguistic experiments have long shown that semantic access occurs routinely without lexical recall. Thus many meanings do not have to be expressed in words. FOK can therefore be conceptually rich and precise (Baars, 1988).

### Mental effort

The sense of mental effort is an important FOK. It commonly occurs in the TOT experience, but also in a range of other tasks that are difficult, slower than expected, require persistence, or involve goal conflict. Duncan and Owen (2000) have done extensive research on this topic, and found that subjective mental effort activates executive regions of the frontal cortex (DL-PFC and ACC) across a variety of tasks. Mental effort may underlie the general intelligence factor *g*, which is found robustly across tests, tasks, and cultures. It may also correspond to the major personality variable persistence, which may explain its cross-cultural relevance, since persistence is needed for high performance in tasks that may be desirable in different cultural contexts. Baumeister has discovered closely related phenomena under the labels “willpower” and “ego depletion.” While these terms have an old-fashioned flavor, they are solidly based in evidence. For example, subjective mental fatigue accumulates during the waking day and may be relieved by increasing self-esteem.

Duncan and Owen (2000) explain subjective effort by a Multiple Demand System (MDS) of the prefrontal cortex. MDS is closely related to dGW, since any region of the C-T system may serve as a binding and broadcasting site according to the argument we have developed.

### Voluntary attention

In a GW perspective, attention is any process that facilitates access to conscious contents, much as eye movements enable access to specific visual events. Visual attention is associated with the eye movement control system, the frontal eye fields and superior colliculi. Cole et al. (2012) have proposed a “cognitive control system” for voluntary attention that involves global connectivity from the prefrontal cortex, and which predicts cognitive control and intelligence. This seems to be another aspect of the mental effort phenomenon discussed above. Goal-driven attention also selects conscious sensory information to recruit, plan, and carry out extended tasks in the world.

The key claim from GW theory regarding dynamics is therefore that conscious contents bind sensory or other cortical-thalamic contents into a winner-take-all coalition, able to broadcast to other regions of the C-T system. No single C-T location is a privileged source.

**Figure 10:**
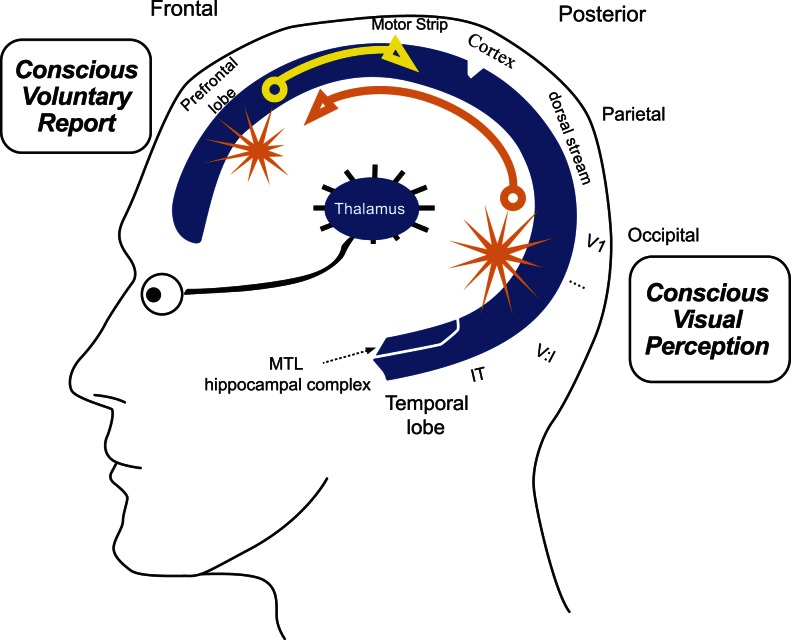
**Perceptual experiences vs. feelings of knowing (FOKs)**. This dGW cartoon shows an occipital broadcast (which must mobilize parietal egocentric and allocentric maps as well) evoking spatiotopic activity in the prefrontal cortex, which is known to initiate voluntary actions (see yellow arrow). Prefrontal activity is shown as a second global workspace burst, consistent with Figure 1. Visuotopic coding is preserved in the dorsolateral prefrontal cortex. The ability to voluntarily report or act upon a spatially specified stimulus follows from this double binding and broadcasting event. Thus, a posterofrontal broadcast is quickly followed by a centrifugal burst from prefrontal regions, including supplementary motor, premotor, and motor cortex. Posterior binding and broadcasting is experienced as a visual event framed in nearby space, while the prefrontal broadcast is a feeling of knowing (FOK) or “fringe” experience (James, 1890).

Figures 1 and 10 (above) showed four hypothetical “binding and broadcasting” sources in cortex, V1 for simple stimuli like a bright star on a dark night, a set of visual areas including IT for object representation at multiple levels, and the MTL/rhinal cortex for event organization.

Prefrontal activation across multiple tasks demanding mental effort (Duncan and Owen, 2000) suggests that sensory conscious experiences are bound and broadcast from the classical sensory regions in the posterior cortex, while voluntary effort, reportable intentions, feelings of effort, and the like, have a prefrontal origin, consistent with brain imaging findings. Figure 10 also suggests that FOK and effort are bound and broadcast from the prefrontal cortex, notably the dorsolateral and anterior cingulate regions, areas that are known to be involved in subjective feelings of effort.

These findings suggests an hypothesis about sensory consciousness compared to “fringe” FOK, feelings of effort, and reportable voluntary decisions. These reportable but “vague” events have been discussed since William James, who gave them equal importance to perceptual consciousness. fMRI studies show that they predominantly involve prefrontal regions, even across tasks that seem very different.

Because of the small-world connectivity of white matter tracts, different integration and distribution hubs may generate different global wave fronts. The sight of a coffee cup may involve an infero-temporal hub signaling to other regions, while the perception of music may emerge from Heschel’s gyrus and related regions. Reportable experiences of cognitive effort might spread outward from a combined DL-PfC/ACC hub.

### Conscious events evoke widespread adaptation or updating

What is the use of binding and broadcasting in the C-T system? One function is to update numerous brain systems to keep up with the fleeting present. GW theory suggested that consciousness is required for non-trivial learning (i.e., learning that involves novelty or significance) (Baars, 1988). While there are constant efforts to demonstrate robust unconscious learning, after six decades of subliminal vision research there is still surprisingly little proof. Subliminal perception may work with known chunks, like facial expressions. While single-word subliminal priming appears to work, Baars (1988) questioned whether novel two-word primes would work subliminally. The subliminal word pair “big house” might prime the word “tall,” while “big baby” might not, because it takes conscious thought to imagine a baby big enough to be called tall. In general, the more novelty is presented, the more conscious exposure is required.

**Figure 11:**
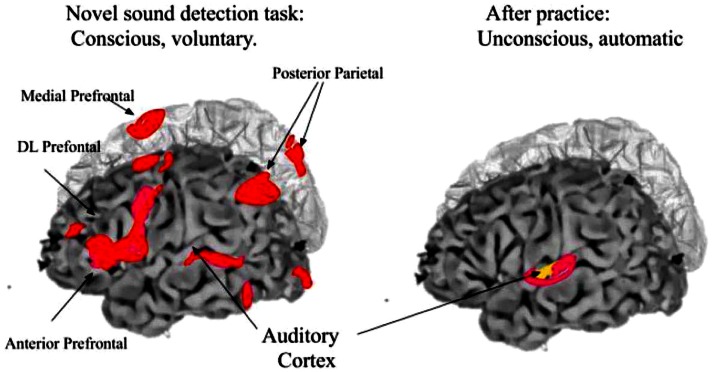
**Cortical adaptation as a novel task becomes automatic**. Schneider (2009) summarized a large literature on habituation of over learned skills. On the left, high BOLD activations are shown in the cognitive control system of the cortex, including the posterior parietal cortex and executive regions of the prefrontal cortex. After the task is practiced to the point of automaticity, only the auditory region of the temporal cortex shows fMRI activity, as required for the auditory detection task. The BOLD signal corresponds to neuronal population activities. These results are consistent with dynamic Global Workspace theory in that the novelty stage involves more conscious and voluntary processing.

It follows that dGW should predict widespread adaptive changes after conscious exposure to an event. That is indeed the consensus for hippocampal-neocortical memory coding (Nadel et al., 2012). However, the hippocampal complex is not currently believed to enable conscious experiences. Nevertheless, episodic memory is by definition “memory for conscious events” (See The Hippocampus and Conscious Contents: a Novel Prediction). Conscious events trigger wide adaptation throughout the C-T system, and in subcortical regions that are influenced by the C-T system. Figure 11 shows how novel, conscious tasks trigger widespread adaptive processing in the C-T core, while the identical task after repetition to the point of automaticity no longer requires high-metabolic C-T processing. Episodic, semantic, and skill (procedural) processing all follow the same curve of high-metabolic processing to novel, conscious learning followed by a drastic drop in conscious access and metabolic (BOLD) activity after learning.

Figure 11 shows an example for auditory search, showing BOLD peaks in many cortical locations, but fading after automaticity due to practice, except in primary auditory cortex. Performance improves as the BOLD signal fades and conscious access recedes (Schneider, 2009). Cortical BOLD fading after training is a robust fact, indicating that conscious (reportable) events evoke widespread adaptation at multiple levels, from single neurons to entire brains (Baars, 1988; Gomez et al., 2009). Adaptation to novelty has been proposed to be one of a small set of necessary conditions for conscious experience (Baars, 1988, Chapter 12).

### Consciously mediated cognition

Most cognitive tasks combine conscious and unconscious elements. If we assume from a GWT perspective that conscious task moments allow novel integrations while unconscious ones implement automatic routines, we can see why fill-in sentences, cued recall, remote associates, “Aha!” experiences and ambiguous stimuli are consciously mediated. Baars and Franklin (2003) suggest that all working memory tasks are consciously mediated – inner rehearsal, executive control, and other WM elements are all claimed to require a conscious cue to execute. If we rehearse seven numbers, some are conscious at any moment, but others are not. We are not aware of non-rehearsed items at any moment in a working memory task, nor of the important role of the basal ganglia in controlling inner speech, or of the automatic (habitual) components of any task. Completely conscious tasks are rare.

Standard tasks in cognitive neuroscience are generally consciously mediated, yet classical notions of cognition give no functional account of that easily observable fact. Standard cognitive tasks like working memory recall could not be done if they were masked by momentary distractions. Apparently the conscious elements of cognitive tasks are often necessary for those tasks to be carried out. Conscious mediation of task elements appears to be mandatory, not optional.

From a GW point of view the answer is straightforward: all tasks that involve unpredictable choice points require global broadcasting to recruit knowledge sources. Baars (1988) devoted several chapters to the near-universality of ambiguity in human cognition, including perception, working memory, memory retrieval, voluntary attention, action planning, and voluntary motor control. When fully predictable tasks are rehearsed to the point of automaticity they tend to drop from consciousness; conversely, when ambiguity is introduced in predictable situations, more conscious involvement is required. The first GW architecture developed by Newell and coworkers was explicitly designed to resolve the multi-level ambiguities of auditory words spoken in a normal noisy space.

Thus GW theory provides a general answer to the question: why are consciously mediated tasks so common? At the same time, it suggests a qualitative answer to the close link between task novelty and conscious access.

## Voluntary Reports of Conscious Events

Conscious contents are routinely assessed by voluntary report, as we know from 200 years of scientific psychophysics. Yet the reason for that fact is far from obvious. Any theory of consciousness must ultimately explain the basic fact that we can voluntarily report an endless range of conscious contents, using an endless range of voluntary actions. GWT suggests the shape of an answer.

Voluntary control is one kind of consciously mediated process. As we learn to ride a bicycle for the first time, each movement seems to come to consciousness. After learning, conscious access drops even as BOLD activity in the C-T core declines (Figure 11). We postulate that conscious involvement is necessary for non-trivial acquisition of knowledge and skills, and that the period of conscious access enables permanent memory traces to be established.

While “verbal report” is the traditional phrase, reports do not have to be verbal – any voluntary response will work. Broca’s aphasics who are cannot speak can point to objects instead. Locked-in (paralyzed) patients, who seem to be comatose, can learn to communicate by voluntary eye movements. Thus “verbal report” should be called “accurate, voluntary report,” using any controllable response.

Voluntary actions can point to objects and events. A “match to sample” task is commonly used to indicate the similarity of two conscious events, and to specify just noticeable differences. Pointing occurs naturally when mammals orient to a novel or significant stimulus. Children develop pointing abilities using “shared attention” in early childhood.

If we assume for simplicity that conscious contents emerge in posterior cortex, and that voluntary actions emerge in frontal and parietal cortex, we can ask the question in dGW terms: how is it that a posterior “binding and broadcasting” event is transformed into a frontally controlled action?

These facts raise the question of how accurate signal transmission occurs between sensory arrays and frontal executive control. In the case of pointing to a single star on a dark night, the physical minimum of light quanta in the retina can be amplified and transmitted to prefrontal cortex, which can control the movement of a single finger to point to the star. Even more remarkably, single neurons in the temporal cortex have been shown to be fired at will in surgical patients using intracranial electrodes, providing only that conscious sensory feedback is given during training (Cerf et al., 2010). Thus the physical minimum to the eye can accurately translate into “any” voluntarily controlled single cell, used as a sensory pointer. Given a million foveal cells for input, and perhaps billions of cortical cells for output, “any-to-any” mapping in the brain can involve remarkably large numbers. With accurate psychophysical performance in both tasks, the signal to noise ratio from receptor to effector cell can approach the physical limit. This precision needs explanation in terms of conscious input and voluntary control.

Figure 10 gives a dGW account of a conscious sensory experience spreading proposes a double broadcast, the sensory one emerging from occipitoparietal regions, and the voluntary one (which may be subjectively effortful) coming from prefrontal executive cortex.

This also suggests an explanation for the standard index of voluntary report. When we report a star on a dark night, posterior broadcasting may lead to frontal binding and ultimately a frontal broadcast. Frontoparietal regents are driven by posterior sensory projections when they become conscious. Because of the striking similarities of spatiotopic coding in frontal and posterior cortices, we can image that sensory consciousness can also trigger a new binding, and broadcast an event in the frontal cortex. Voluntary action is therefore an extension of GW dynamics.

**Figure 12:**
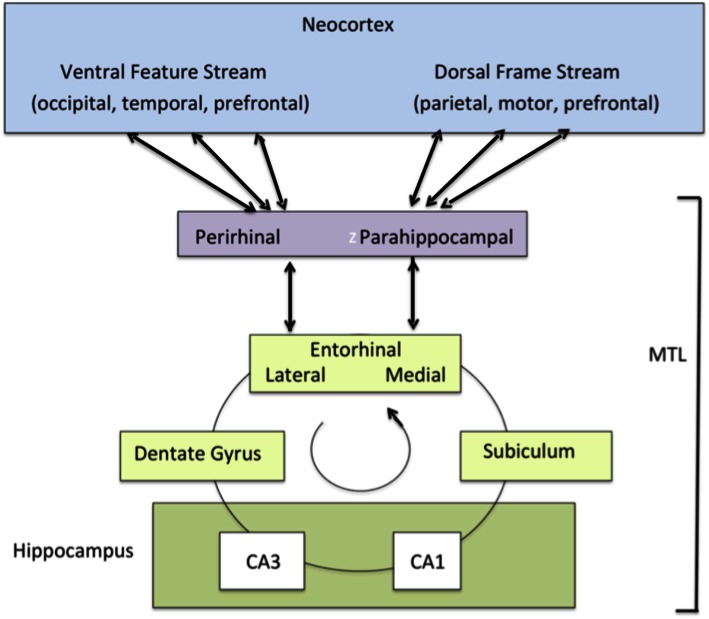
**Hippocampal-neocortical binding and broadcasting**. The hippocampal complex in relation to the neocortex. Notice that the flow of information streams in both directions, with the interesting exception of the subiculum. Thus resonant adaptation can take place in almost all regions of the hippocampal-neocortical complex. This region appears as a major convergence and broadcasting zone, and has been proposed to combine the dorsal and ventral streams of visual cortex (Shimamura, 2010). Direct MTL neuronal recording shows responding to conscious, but not unconscious visual input. Phylogenetically, the hippocampus is ancestral to the neocortex, and controls a fully autonomous sensorimotor brain.

Figure 12 shows an intracranial electrode grid recording of the left hemisphere of an epileptic patient with implanted cortical electrodes for presurgical testing. It shows a word being perceived consciously, and after a suitable delay, being reported. The dependent variable in Figure 12 is gamma synchrony bursts in four different frequency ranges (67–117 Hz).

For the first time in history we are able to look at the cortex in real time and with high spatial resolution as the patient hears a spoken word and then reports it. In both conditions, hearing and reporting, we can see phase coupling bursts in the left LIFG (lateral inferior frontal gyrus), Broca’s area. A dGW interpretation would suggest a broadcast from auditory cortex in the first condition (a), spreading to Broca’s area and setting up adaptive resonance lasting for several seconds (about the duration of working memory, 10–30 s). When the patient repeats the word in part (b) the relevant speech production regions are already primed by the stimulus broadcast. This account is still sketchy, since we only see two snapshots in time. However, we can use dGW to make more precise and testable predictions.

## The Hippocampus and Conscious Contents: A Novel Prediction

Hippocampus is generally thought to encode episodic memories but not conscious experiences. This is puzzling, since episodic memories come from conscious episodes in the first place. It would be odd if these twin functions were not linked.

Because the neocortical-thalamic system arose with mammals some 200 million years ago, some kinds of consciousness appear to be evolutionarily ancient. Neurobiologists are increasingly searching for brain homologs and analogs of the C-T system found in mammals. What G. M. Edelman calls “higher level” (symbolic) consciousness would be based on the frontal expansion of the primate brain, giving rise to novel symbolic and language-dependent capacities.

In humans there is no anatomical or physiological barrier between paleocortex and neocortex. We therefore propose, as an hypothesis, that like neocortex, the hippocampal complex helps to specify conscious experiences as well as encoding episodic memories. dGW proposes that any region of neocortex can function as a source or receiving region for conscious experiences. We would therefore add “paleocortex,” including the hippocampus, to “neocortex.” What may be distinctive about the hippocampal complex is its role in encoding and decoding of conscious episodes into widely distributed memory traces. There is direct evidence from brain recordings in the MTL (Panagiotaropoulos et al., 2012), which follow the conscious percept in a binocular rivalry task, rather than the physical stimulus.

This seems to be contradicted by the classical case of patient HM, a rare case of very precise surgical excision of the bilateral MTL, which contains the hippocampal complex. HM appeared to have normal conscious perception, while his episodic memory was lost. HM’s surgery therefore seemed to dissociate conscious perception from memory. HM was studied over a 60-year period and is still an important source of insight.

Cortical impairments are often difficult to interpret functionally, because patients spontaneously reorganize their behavior and cortex to compensate. For example, cutting the corpus callosum destroys about 200 million axons linking the hemispheres, but patients learn to use cross-body arm and eye movements to compensate. Early split brain patients seemed to show no impairments at all; careful experimental tests later on showed the precise nature of their functional losses. A similar debate occurred about the results of prefrontal lobotomy, stroke, minimal brain damage, and the like.

## Summary

A GW is a functional hub of signal binding and propagation in a population of loosely coupled signaling agents. Neurons and neuronal cell assemblies can be defined as such agents when they respond selectively to input.

Conscious experiences may reflect a GW function in the brain. The brain has many anatomical hubs, but conscious percepts are unitary and internally consistent at any given moment. This suggests that a brain-based GW capacity cannot be limited to only one anatomical hub. Rather, it should be sought in a dynamic and coherent binding capacity – a *functiona*l hub – for neural signaling over multiple networks. A number of findings are consistent with the theory.

In humans the C-T complex underlies reportable conscious percepts, concepts, FOK, visual images and executive functions. While subcortical areas are often sometimes proposed to specify conscious contents, the human evidence is slight and disputed. Because cortex and thalamus are interleaved so densely as to constitute a single functional system, we will refer here to the C-T system as a whole. C-T pathways permit constant reentrant signaling, so that multiple spatiotopic maps can sustain or inhibit each other. The daily states of the core are controlled by basal brain nuclei.

Global workspace theory follows the historic distinction between the “focus” of experience vs. the largely implicit background of experience. Extensive evidence shows that visual and auditory consciousness flows from the respective sensory cortices to frontoparietal regions. This directionality differentiates GW dynamics from information integration theory and dynamic core theory.

Cortico-thalamic core is a great mosaic of multi-layered two-dimensional neuronal arrays. Each array of cell bodies and neurites projects to others in topographically systematic ways. Since all C-T pathways are bidirectional, signaling is “adaptively resonant” (reentrant). In this complex, layered two-dimensional arrays are systematically mirrored between cortex and thalamus, region by region.

The C-T nexus appears to be the most parallel-interactive structure in the brain, allowing for efficient signal routing from any neuronal array to any other. This connectivity is different from other structures that do not directly enable conscious contents, like the cerebellum. The cerebellum is organized in modular clusters that can run independently of each other, in true parallel fashion. But in the C-T core any layered array of cortical or thalamic tissue can interact with any other, more like the world-wide web than a server farm.

Cortico-thalamic pathways run in all canonical directions and follow small-world organization, so that each array is efficiently linked to many others. The entire system acts as an oscillatory medium, with markedly different global regimes in conscious and unconscious states.

Global workspace dynamics interprets the traditional distinction between the “object” and “ground” of experiences as a directional flow between the moment-to-moment focus of conscious experience vs. the implicit background and sequelae of focal contents. The proposed directionality of broadcasting suggests a testable distinction with information integration theory and dynamic core theory.

For example, while sensory experiences are proposed to bind and broadcast from posterior cortex, “fringe conscious” FOK plausibly emerge from non-sensory cortices, and may therefore broadcast rostrocaudally.

Finally, dGW suggests the counter-intuitive idea that in intact humans, the MTL supports conscious episodes. The classic lesion case of HM seems to contradict that idea, but traditional psychophysical testing does not show episodic organization. We therefore suggest that the positive evidence from brain recording methods for conscious experience in MTL may outweigh lesion evidence against it, given the well-established tendency toward cortical reorganization after injury.

## Conflict of Interest Statement

The authors declare that the research was conducted in the absence of any commercial or financial relationships that could be construed as a potential conflict of interest.

## Footnotes

^2^Previous work is available at Cognitive Computing Research Group, The University of Memphis (http://ccrg.cs.memphis.edu/papers.html) Steriade (2006) we use “C-T” to emphasize the driving role of the cortex.

^3^See http://en.wikipedia.org/wiki/Pointillism

^4^To avoid confusion, this paper uses the word “frame” instead of “context” for unconscious constraints on conscious contents (Baars, 1988).

^5^The LIDA model suggests that cognitive processes involve cascading action-perception cycles (Franklin et al., 2012), called cognitive cycles in the model. After filtering for salience, each cognitive cycle produces a global broadcast that both facilitates several modes of learning and recruits resources for consciously mediated action selection. During the preconscious understanding phase of the cognitive cycle, the current situational model (Franklin et al., 2012), acting as a blackboard model, is updated by accessing previously learned information from sensory memory, recognition memory, spatial memory, episodic memory and semantic memory. The next contents of consciousness selects itself dynamically from cognitive structures whose building blocks are the accessed cognitive representations. Thus consciousness creates access leading to conscious thoughts and subsequent actions.

^6^The term “oscillation” is often used to describe wave-like activity in the brain. However, Freeman (2007) points out that there is no proof that wave-like activity patterns return to their origins, like mathematical sine waves. The notion of reentry and adaptive resonance indeed suggests the opposite.

^7^Major thalamic divisions are called “nuclei,” but they are typically layered arrays of neurons that are tightly folded, so that they appear as clumps to the naked eye.

## Appendix

### Basic evidence

1. Raw EEG signature: conscious states show irregular, low-amplitude, and fast EEG activity 12–200 Hz, using both intracranial and surface measures. Unconscious states tend to show regular, high-amplitude, and slow voltages at less than 4 Hz.
2. Cortex and thalamus: consciousness depends on the thalamocortical complex, turned on and off by brainstem neuromodulation. Cortical regions are bidirectionally linked to corresponding layered arrays in thalamus.
3. Widespread brain activity: conscious contents are associated with widespread brain activation related to the content. Unconscious stimulation evokes only local cortical activity. Conscious scenes also involve wide effects outside the focus of current conscious contents, as indicated by implicit learning, episodic memory, biofeedback training of autonomic and motor function, and the like.
4. Wide range of contents: consciousness has an extraordinary range of different contents – perception in the various senses, endogenous imagery, emotional feelings, inner speech, concepts, action-related ideas and “fringe” experiences such as feelings of familiarity.
5. Informativeness: consciousness may fade when signals become redundant; a loss of information may also lead to a loss of conscious access. Studies of attentional selection also show a strong preference for more informative conscious stimuli.
6. The rapidly adaptive and fleeting nature of conscious events: immediate experience of the sensory past may last a few seconds, and our fleeting present is perhaps 10–30 s, the duration of working memory when arbitrary material can be accessed at will. In contrast, vast bodies of unconscious knowledge reside in long-term memory.
7. Internal consistency: consciousness is marked by a consistency constraint. For example, while multiple meanings of most words are active for a brief time after presentation, only one becomes conscious at any moment. In general, of two mutually inconsistent stimuli presented simultaneously, only one can become conscious.
8. Limited capacity and seriality: the capacity of consciousness at any given moment seems limited to one consistent scene (see above). The flow of such scenes is serial, in contrast with the massive parallelism of the brain as observed directly.
9. Sensory binding: the sensory brain is functionally segregated such that different cortical areas are specialized to respond to different features such as shape, color, or object motion. One classic question is how these functionally segregated regions coordinate their activities in order to generate the gestalts of ordinary conscious perception.
10. Self-attribution: conscious experiences are always attributed to an experiencing self, the “observing self” as James called it. Self-functions appear to be associated with midline regions of cortex in humans.
11. Accurate reportability: conscious contents are reportable by a wide range of voluntary responses, often with very high accuracy. The standard operational index of consciousness is based on accurate reportability.
12. Subjectivity and privacy: consciousness is marked by the existence of a private flow of events available only to the experiencing subject, though much of it is available for public report.
13. Focus-fringe structure: while consciousness tends to be thought of as a focal, clearly articulated set of contents, “fringe conscious” events, like feelings of familiarity, the TOT experience, etc., are also important.
14. Facilitation of learning: there is very little evidence for long-term learning of unconscious input. In contrast, the evidence of learning of conscious episodes is overwhelming. Even implicit learning requires conscious attention to the stimuli from which implicit regularities are (unconsciously) inferred.
15. Stability of contents: conscious contents are impressively stable, given the variability of input that is dealt with. Even abstract contents such as beliefs, concepts, and the motivational self are remarkably stable over years.
16. Allocentricity: neural representations of external objects make use of diverse frames of reference. Conscious scenes, generally speaking, have allocentric character, though they are shaped by egocentric and other unconscious frameworks.
17. Conscious knowing and decision making: consciousness is obviously useful for knowing the world around us, as well as for knowing certain of our own internal processes. Conscious intentionality may be particularly well suited for voluntary decision making.
18. Orientation to time, place, self, and other.
19. Simple working memory.
20. Intermediate recall.
21. Attention, mental effort, and calculation.
22. Simple language, such as naming common objects.
23. Basic visuomotor skills.
